# COVID-19 Severity and COVID-19–Associated Deaths Among Hospitalized Patients with HIV Infection — Zambia, March–December 2020

**DOI:** 10.15585/mmwr.mm7022a2

**Published:** 2021-06-04

**Authors:** Duncan Chanda, Peter A. Minchella, Davies Kampamba, Megumi Itoh, Jonas Z. Hines, Sombo Fwoloshi, Mary Adetinuke Boyd, Kalongo Hamusonde, Lameck Chirwa, Kotey Nikoi, Robert Chirwa, Mpanji Siwingwa, Suilanji Sivile, Khozya D. Zyambo, Aggrey Mweemba, Nyuma Mbewe, Katongo H. Mutengo, Kennedy Malama, Simon Agolory, Lloyd B. Mulenga

**Affiliations:** ^1^Zambian Ministry of Health, Lusaka, Zambia; ^2^CDC, Lusaka, Zambia.

The effect of HIV infection on COVID-19 outcomes is unclear. Studies in South Africa (*1*) and the United Kingdom (*2*) found an independent association between HIV infection and COVID-19 mortality; however, other studies have not found an association between poor COVID-19 outcomes and either HIV status among hospitalized patients (*3*–*5*) or HIV-associated factors such as CD4 count, viral load, or type of antiretroviral therapy (ART) (*6*). The effect of HIV infection on COVID-19 outcomes remains an urgent question in sub-Saharan Africa, where many countries are experiencing dual HIV and COVID-19 epidemics, and capacity to treat severe COVID-19 is limited. Using data from patients with probable or confirmed COVID-19 admitted to specialized treatment centers during March–December 2020 in Zambia, the Zambian Ministry of Health and CDC assessed the relationship between HIV infection and severe COVID-19 and COVID-19–associated death. Among 443 patients included in the study, 122 (28%) were HIV-positive, and of these, 91 (89%) were receiving ART at the time of hospitalization. HIV status alone was not significantly associated with severe COVID-19 at admission or during hospitalization or with COVID-19–associated death. However, among HIV-positive persons, those with severe HIV disease were more likely to develop severe COVID-19 and were at increased risk for COVID-19–associated death. Ensuring that persons maintain HIV disease control, including maintaining ART continuity and adherence, achieving viral suppression, and addressing and managing underlying medical conditions, could help reduce COVID-19–associated morbidity and mortality in sub-Saharan Africa.

Zambia is a landlocked country in southeastern Africa, with an estimated population of 17.4 million* and a generalized HIV/AIDS epidemic with HIV prevalence among persons aged ≥15 years of 12.1% (*7*). Beginning in March 2020, patients with a diagnosis of probable or laboratory-confirmed COVID-19 in Zambia were admitted to one of five Zambia Ministry of Health specialized COVID-19 treatment centers located in the capital city of Lusaka (two treatment centers) and in Ndola, Kabwe, and Livingstone. Confirmed cases were those with positive reverse transcription–polymerase chain reaction or rapid antigen test results for SARS-CoV-2, the virus that causes COVID-19. Patients with diagnosed probable cases had radiologic evidence suggestive of COVID-19 with acute respiratory symptoms. Treatment centers have specifically designated isolation and treatment units staffed by clinicians and nurses trained in COVID-19 clinical management. All patients who received medical care for COVID-19 in these centers during March–December 2020 and who provided verbal consent to receive treatment were enrolled in the COVID-19 clinical outcomes study. Patient demographic, clinical, and survival time data were collected during hospitalization until patients died or were discharged. Data were primarily collected electronically in real time by trained staff members using a standardized case record form^†^; among patients who had received COVID-19 care before the start of data collection, medical records were reviewed and abstracted into the case record form. Study staff members contacted discharged patients by telephone 28 days after admission to determine their health status. 

Severe COVID-19 was defined as having an oxygen saturation <90%, respiratory rate >30 breaths/minute, or a need for oxygen therapy.^§^ COVID-19 severity was assessed at admission and during hospitalization. HIV status was self-reported, and patients with unknown status or deemed eligible and consented for testing were tested at admission. Underlying medical conditions were self-reported and included cardiac disease, hypertension, diabetes, other pulmonary disease, active tuberculosis (TB), previous TB, asthma, kidney disease, liver disease, neurologic disorder, asplenia, malignant neoplasm, and current smoking. The number of underlying conditions was summed for each patient. Patients with severe HIV disease were defined as those meeting one or more of the following criteria: 1) severely anemic (hemoglobin <8.0 g/dL); 2) CD4 <200 cells/*μ*L; 3) active TB, including patients taking anti-TB medication; or 4) underweight (body mass index [BMI] <18.5 kg/m^2^). HIV-positive patients who did not meet any of the conditions were considered to have controlled HIV infection.

The three primary outcomes assessed in the study were 1) severe COVID-19 at admission; 2) severe COVID-19 during hospitalization; and 3) death. Mixed-effects logistic regression models were used to assess associations between exposure variables (age, sex, number of underlying conditions, and HIV status) and the two severe COVID-19 outcomes (at admission and during hospitalization). Mixed-effects Cox proportional hazards regression models were used to examine time to COVID-19–associated death in relation to exposure variables. All models included a random-effects term for treatment center, and other covariates were sex, age, and number of underlying health conditions. Cox models were also adjusted for COVID-19 severity at admission. Similar mixed-effects logistic regression and mixed-effects Cox proportional hazards models were used to assess COVID-19 outcomes among HIV-positive persons stratified by HIV infection control status; covariates in these models included sex, age, and treatment center (random effects term). Data were analyzed using R (version 4.0.2; R Foundation). An alpha level of 0.05 was used to assess statistical significance. The study protocol was approved by the University of Zambia Biomedical Research Ethics Committee, reviewed by CDC, and conducted consistent with applicable federal law and CDC policy.^¶^

Among 612 hospitalized patients who were eligible for the study, 443 (72%) had HIV status recorded. Among those patients, 122 (28%) were HIV-positive, and among the 102 HIV-positive persons who provided information on ART status, 91 (89%) were receiving ART (Table 1). Although sex and mean age did not differ by HIV status, among HIV-negative patients, the proportion of those aged ≥60 years was higher than the proportion of those aged <60 years (p = 0.002). HIV-positive patients also were more likely to be anemic (defined as hemoglobin [Hb] <12 g/dL for women and Hb <13 g/dL for men) (p<0.001) or severely anemic (Hb <8 g/dL) (p = 0.004) and to report having two or more underlying medical conditions than were HIV-negative patients (p = 0.017).

**TABLE 1:** Demographic and clinical characteristics of persons hospitalized for confirmed or probable COVID-19 (N = 443),* by HIV status— Zambia, March–December 2020

Characteristic	No. (%)		p-value^†^
	HIV-negative (n = 321)	HIV-positive (n = 122)	
Confirmed SARS-CoV-2 infection^§^	238 (74)	87 (71)	0.69
**Demographic**			
Male	190 (59)	64 (52)	0.24
Mean age, yrs (SD)	48.9 (18.1)	46.4 (12.9)	0.11
<15	4 (1)	0 (—)	
15–34	71 (22)	21 (17)	
35–49	99 (31)	53 (43)	
50–59	46 (14)	28 (23)	
≥60	101 (31)	20 (16)	0.002
**HIV-positive patient indicator of disease control**			
On ART^¶^	N/A	91 (89)**	—
VL <1,000 copies of HIV RNA/mL	N/A	24 (86)**	—
CD4 ≥200 cells/*μ*L	N/A	16 (50)**	—
Anemia^††^	84 (37)**	49 (69)**	<0.001
Underweight (BMI <18.5 kg/m^2^)	12 (6)**	10 (15)**	0.059
**Laboratory value**			
Severe anemia^§§^	10 (4)**	11 (15)**	0.004
Median WBC (IQR)	7.42 (5.12–11.1)	7.32 (4.5–12)	0.55
WBC <4 IU	23 (10)**	11 (16)**	0.25
CRP >30 mg/L	62 (51)**	28 (62)**	0.26
**Underlying medical condition^¶¶^**			
None	154 (48)	54 (44)	0.017^†^
1	101 (31)	29 (24)	
≥2	66 (21)	39 (32)	
Diabetes	46 (14)	18 (15)	1.00
Hypertension	114 (36)	32 (26)	0.081
Active tuberculosis	5 (2)	16 (13)	<0.001
**Outcome**			
Severe COVID-19*** at admission	176 (55)	62 (51)	0.52
Severe COVID-19*** during hospitalization	188 (59)	68 (56)	0.52
Died	61 (19)	17 (14)	0.27

**Abbreviations:** ART = antiretroviral therapy; BMI = body mass index; CRP = C-reactive protein; Hb = hemoglobin; IQR = interquartile range; IU = international units; N/A = not applicable; SD = standard deviation; TB = tuberculosis; VL = viral load; WBC = white blood cell count.

* Reported as number (%) unless indicated otherwise. Denominator is total number by HIV status unless indicated otherwise. HIV status was self-reported and confirmed by HIV test at hospital admission (if the patient specifically consented to an HIV test).

^†^ P-values <0.05 were considered statistically significant. Comparison of proportions was via chi-square test, comparison of normally distributed variables was via Welch’s two sample t-test, and comparison of nonnormally distributed variables was via Wilcoxon test. P-values for age ≥60 years and two or more comorbidities are for binary variables (i.e., <60 versus ≥60 years and less than two versus two or more comorbidities).

^§^ COVID-19 cases were confirmed via SARS-CoV-2 reverse transcription–polymerase chain reaction or SARS-CoV-2 antigen detecting rapid diagnostic test. Probable cases had acute respiratory symptoms with radiological evidence that was suggestive of COVID-19.

^¶^ ART status was self-reported.

** Denominator was less than the total number by HIV status.

^††^ Anemia was defined as Hb <12 g/dL for women and Hb <13 g/dL for men.

^§§^ Severe anemia was defined as Hb <8 g/dL.

^¶¶^ Comorbidities composite term is based on the number of self-reported comorbidities. Eligible comorbidities included chronic cardiac disease, hypertension, pulmonary disease, active TB, previous TB, asthma, kidney disease, liver disease, neurologic disorder, diabetes, current smoking, asplenia, and malignant neoplasm. Conditions commonly linked to COVID-19 severity (diabetes and hypertension) and related to HIV (active TB) are listed in the table.

*** Severe COVID-19 was defined as one or more of the following conditions: oxygen saturation <90%, respiratory rate >30 breaths/minute, and need for oxygen therapy.

Age ≥60 years and having two or more underlying medical conditions were associated with severe COVID-19; however, HIV status alone was not associated with severe COVID-19 at admission or during hospitalization (Table 2). Similarly, male sex, age ≥60 years, and reporting two or more underlying medical conditions were significantly associated with COVID-19–associated death, but HIV status was not. However, among HIV-infected patients, compared with patients with controlled HIV, severe HIV disease was associated with severe COVID-19 at admission (adjusted odds ratio [aOR] = 3.91; 95% confidence interval [CI] = 1.69–9.69) or during hospitalization (aOR = 4.42; 95% CI = 1.83–11.66) and with increased COVID-19–associated death (aOR = 3.27; 1.21–8.79).

**TABLE 2:** Factors* associated with severe COVID-19^†^ at hospital admission, severe COVID-19 during hospitalization, and COVID-19–associated death among hospitalized patients with HIV infection^§^ — Zambia, March–December 2020

Factor	aOR (95% CI)		aHR (95% CI)
	Severe COVID-19 at admission	Severe COVID-19 during hospitalization	COVID-19–associated death
Male sex	1.31 (0.92–1.87)	1.20 (0.84–1.71)	1.71 (1.07–2.76)
Age ≥60 yrs	2.64 (1.72–4.03)	3.10 (2.01–4.83)	2.09 (1.32–3.29)
Two or more underlying medical conditions*	2.79 (1.71–4.56)	2.57 (1.57–4.29)	1.78 (1.11–2.83)
HIV-positive^¶^	0.92 (0.59–1.46)	1.00 (0.63–1.57	0.88 (0.49–1.56)
Controlled HIV (n = 85)	Ref	Ref	Ref
Severe HIV disease (n = 37)^¶^	3.91 (1.69–9.69)	4.42 (1.83–11.66)	3.27 (1.21–8.79)

**Abbreviations**: aHR = adjusted hazard ratio; aOR = adjusted odds ratio; BMI = body mass index; CI = confidence interval; Ref = referent; TB = tuberculosis.

* Underlying medical conditions included cardiac disease, hypertension, diabetes, other pulmonary disease, active TB, previous TB, asthma, kidney disease, liver disease, neurologic disorder, asplenia, malignant neoplasm, and current smoking.

**^†^** Oxygen saturation <90%, respiratory rate >30 breaths/minute, or need for oxygen therapy.

**^§^** Mixed-effects logistic regression models were used for severe COVID-19 outcomes. Models were adjusted for sex, age (<60 and ≥60 years), comorbidities (fewer than two and two or more), and treatment center (random effects term). Cox proportional hazards models were used for COVID-19–associated death outcome. Models were adjusted for sex, age (<60 and ≥60 years), underlying medical conditions (fewer than two and two or more), COVID-19 severity at admission (mild and severe) and treatment center (random effects term). Reference categories for exposure variables in all models were female sex, age <60 years, fewer than two underlying medical conditions, and HIV-negative status.

^¶^ HIV-positive patients were classified as having severe HIV disease if they met one or more of the following conditions: severely anemic (<8.0 g/dL), CD4 <200 cells/*μ*L, active TB, and underweight (BMI <18.5 kg/m^2^).

## Discussion

HIV infection was not independently associated with worse outcomes among patients hospitalized for COVID-19 in Zambia. This finding is consistent with results from smaller studies among hospitalized patients in North America (*3*), Europe (*4*), and South Africa (*5*). However, among HIV-positive patients hospitalized for COVID-19, those with severe HIV disease were more likely to develop severe COVID-19 or to die of COVID-19 compared with those with controlled HIV disease. Ensuring that HIV-positive persons maintain disease control, including sustaining ART continuity and adherence, achieving viral suppression (<1,000 copies of HIV RNA per mL), and addressing underlying medical conditions, could reduce COVID-19–associated morbidity and mortality in sub-Saharan Africa, including Zambia.

The relationship between severe HIV disease and poor COVID-19 outcomes underscores the importance of Zambia’s progress toward ending the HIV epidemic and of efforts to maintain HIV services during the COVID-19 pandemic. As of June 2020, approximately 90% of an estimated 1.2 million HIV-positive Zambians were receiving ART, and nearly 90% of those patients were virally suppressed (*7*). In addition, since 2019, approximately 350,000 HIV-positive Zambians have completed a course of TB preventive treatment.** Since the first COVID-19 cases were detected in Zambia in March 2020, the national HIV program has made a concerted effort to continue to identify persons with new HIV infections and initiate ART as part of routine HIV case management. To ensure that all patients receiving ART have safe and uninterrupted access to treatment, the national program also took steps to accelerate dispensation of multimonth ART prescriptions for stable patients (K Mweebo, CDC, unpublished data, 2021). These efforts might have helped some HIV-positive patients adhere to ART and possibly avoid more severe COVID-19 as well as complications from HIV. However, there is still much that remains unknown about the impact of COVID-19 on persons living with HIV infection.

The findings in this report are subject to at least three limitations. First, the small sample size might have contributed to the absence of a significant association between HIV status and COVID-19 outcomes. Whereas cohort studies with larger populations in South Africa (*1*) and the United Kingdom (*2*) reported that HIV-positive persons were at increased risk for COVID-19–associated death, smaller studies among hospitalized patients (including the current study) have not (*3*–*5*). Second, differences in findings between these two types of studies might also be attributable to collider bias (wherein HIV and COVID-19 might independently lead to hospitalization, distorting the association between the conditions) (*8*), which might also limit the generalizability of these findings beyond hospitalized patients. Finally, as with many studies conducted during emergency responses, data completeness was a limitation because clinicians who were responsible for data collection were also responding to other urgent demands at the COVID-19 treatment centers. Approximately one quarter of eligible patients were excluded from the study because critical information about them was missing; moreover, some data, including CD4 counts and HIV viral load testing results, were sparse among included patients.

The findings from this study indicate that HIV-positive persons with severe HIV disease appear to be more likely to develop severe COVID-19 and die of COVID-19 than those with controlled HIV infections. In Zambia and other sub-Saharan African countries with high HIV prevalence and limited capacity to treat severe COVID-19, continued efforts to ensure that HIV-positive persons maintain control of their HIV infections through retention in care and adherence to ART and by addressing and managing their underlying medical conditions, could help limit COVID-19–associated morbidity and mortality and HIV-associated morbidity. Larger studies that include more robust data on CD4 counts and viral loads might provide a more nuanced picture of the potential impact of HIV disease status on COVID-19–associated morbidity and mortality.

**Summary**

**What is already known about this topic?**

The effect of HIV infection on COVID-19 outcomes is unclear.

**What is added by this report?**

HIV infection was not associated with poor outcomes among patients hospitalized for COVID-19 in Zambia. However, HIV-positive patients with severe HIV disease were more likely to develop severe COVID-19 or die of COVID-19.

**What are the implications for public health practice?**

Ensuring that persons maintain HIV disease control, including by maintaining ART treatment continuity and adherence, achieving viral suppression, and addressing underlying medical conditions, could help reduce COVID-19–associated morbidity and mortality in sub-Saharan Africa.

## Footnotes

*
https://www.cia.gov/the-world-factbook/static/16a49aca1982c8b59274c8eb0cf6ff0b/ZA-summary.pdf

†
https://www.who.int/teams/health-care-readiness-clinical-unit/covid-19/data-platform

§ https://apps.who.int/iris/rest/bitstreams/1328457/retrieve

¶ 45 C.F.R. part 46.102(l)(2), 21 C.F.R. part 56; 42 U.S.C. Sect. 241(d); 5 U.S.C. Sect. 552a; 44 U.S.C. Sect. 3501 et seq.

** U.S. President’s Emergency Plan for AIDS Relief data for Zambia. https://data.pepfar.gov/
